# Temporal Decline in Intravascular Albumin Mass and Its Association with Fluid Balance and Mortality in Sepsis: A Prospective Observational Study

**DOI:** 10.3390/jcm14155255

**Published:** 2025-07-24

**Authors:** Christian J. Wiedermann, Arian Zaboli, Fabrizio Lucente, Lucia Filippi, Michael Maggi, Paolo Ferretto, Alessandro Cipriano, Antonio Voza, Lorenzo Ghiadoni, Gianni Turcato

**Affiliations:** 1 Institute of General Medicine and Public Health, Claudiana College of Health Professions, 39100 Bolzano, Italy; 2 Health Professions Management, South Tyrolean Health Authority (SABES-ASDAA), 39100 Bolzano, Italy; zaboliarian@gmail.com; 3 Intermediate Care Unit, Department of Internal Medicine, Hospital Alto Vicentino (AULSS7), 36100 Vicenza, Italy; fabrizio.lucente@aulss7.veneto.it (F.L.); lucia.filippi@aulss7.veneto.it (L.F.); michael.maggi@aulss7.veneto.it (M.M.); paolo.ferreto@aulss7.veneto.it (P.F.); 4 Emergency Department, Nuovo Santa Chiara Hospital, Azienda Ospedaliero-Universitaria Pisana, 56100 Pisa, Italy; alessandrocipriano@gmail.com; 5 Emergency Medicine Residency Program, Department of Emergency Medicine, Humanitas University Hospital, 20089 Rozzano, Italy; antonio.voza@humanitas.it; 6 Department of Clinical and Experimental Medicine, University of Pisa, 56126 Pisa, Italy; lorenzo.ghiadoni@unipi.it; 7 Department of Health Sciences, UniCamillus-Saint Camillus International University of Health Sciences, 00131 Rome, Italy

**Keywords:** sepsis, intravascular albumin mass, capillary leak syndrome, fluid resuscitation, mortality prediction

## Abstract

**Background**: Intravascular albumin mass represents the total quantity of albumin circulating within the bloodstream and may serve as a physiologically relevant marker of vascular integrity and fluid distribution in sepsis. While low serum albumin levels are acknowledged as prognostic indicators, dynamic assessments based on albumin mass remain insufficiently explored in patients outside the intensive care unit. **Objectives**: To describe the temporal changes in intravascular albumin mass in patients with community-acquired sepsis and to examine its relationship with fluid balance and thirty-day mortality. **Methods**: This prospective observational study encompassed 247 adults diagnosed with community-acquired sepsis who were admitted to a high-dependency hospital ward specializing in acute medical care. The intravascular albumin mass was calculated daily for a duration of up to five days, utilizing plasma albumin concentration and estimated plasma volume derived from anthropometric and hematologic data. Net albumin leakage was defined as the variation in intravascular albumin mass between consecutive days. Fluid administration and urine output were documented to ascertain cumulative fluid balance. Repeated-measures statistical models were employed to evaluate the associations between intravascular albumin mass, fluid balance, and mortality, with adjustments made for age, comorbidity, and clinical severity scores. **Results**: The intravascular albumin mass exhibited a significant decrease during the initial five days of hospitalization and demonstrated an inverse correlation with the cumulative fluid balance. A greater net leakage of albumin was associated with a positive fluid balance and elevated mortality rates. Furthermore, a reduced intravascular albumin mass independently predicted an increased risk of mortality at thirty days. **Conclusions**: A reduction in intravascular albumin mass may suggest ineffective fluid retention and the onset of capillary leak syndrome. This parameter holds promise as a clinically valuable, non-invasive indicator for guiding fluid resuscitation in cases of sepsis.

## 1. Introduction

Sepsis is a critical condition characterized by life-threatening organ dysfunction resulting from a dysregulated host response to infection. Despite advancements in critical care, sepsis continues to be a significant cause of morbidity and mortality globally [1,2]. A central pathophysiological aspect of sepsis is the increased permeability of the endothelium, which contributes to capillary leak syndrome and the compromise of vascular barrier integrity [3]. In this context, albumin, the most abundant circulating protein and a key determinant of intravascular oncotic pressure, serves a dual function: as a biomarker of vascular integrity and as a mediator of intravascular volume homeostasis [4].

In healthy individuals, approximately 40% of total body albumin is located in the plasma, with the remaining 60% distributed in the interstitial space. Albumin continuously exchanges across the capillary wall through a tightly regulated process involving the endothelial glycocalyx and intercellular junctions [5]. However, during sepsis, systemic inflammation disrupts these regulatory mechanisms, leading to significantly elevated transcapillary escape rates of albumin [6]. This process results in a rapid decline in intravascular albumin mass (IAM), which subsequently reduces oncotic pressure, exacerbates fluid extravasation, and contributes to the vicious cycle of fluid overload and tissue edema [7]. The net albumin leakage (NAL) from the vascular to the interstitial space may thus represent a quantifiable marker of capillary dysfunction in sepsis.

The prognostic significance of hypoalbuminemia in sepsis has been extensively documented. Numerous studies have demonstrated that low serum albumin concentrations upon admission are independently associated with increased short-term mortality, even after adjusting for disease severity scores such as SOFA or APACHE II, and including Intermediate Care Units (IMCUs) [8]. Beyond static measurements, recent research underscores the importance of dynamic trends in albumin levels during early sepsis resuscitation [9]. Persistent or worsening hypoalbuminemia during the first 72 h is strongly associated with poor clinical outcomes, including acute kidney injury, prolonged mechanical ventilation, and death. Conversely, stabilization or recovery of albumin levels appears to indicate vascular recovery and clinical improvement [10].

Fluid resuscitation, a fundamental aspect of early sepsis management, introduces additional complexity to this dynamic. While prompt fluid administration can restore perfusion, excessive or prolonged fluid loading in the context of capillary leak may paradoxically exacerbate edema and organ dysfunction. An increasing body of evidence suggests that a positive cumulative fluid balance is independently associated with poorer outcomes in septic patients [11]. This has led to a growing interest in fluid stewardship and the utilization of biomarkers to guide resuscitation. The trajectory of IAM, particularly when adjusted for fluid dilution effects and calculated using mass-based approaches, may offer valuable insights into the effectiveness of fluid therapy and the evolving vascular status of the patient [12].

Despite these insights, most studies examining albumin in sepsis have concentrated on intensive care settings or evaluated serum concentrations rather than actual mass-based measures [13]. The clinical relevance of IAM trajectories in intermediate care settings, where patients are often at a critical juncture between stability and deterioration, remains underexplored. Notably, intermediate care units (IMCUs) provide a unique environment to observe early pathophysiological changes before full-blown organ failure necessitates ICU transfer [14,15].

This prospective observational study aimed to address this knowledge gap by characterizing the temporal trends in IAM among patients with community-acquired sepsis admitted to a high-intensity IMCU. The specific investigation included (1) the dynamic changes in IAM over the first five days of hospitalization, (2) the relationship between IAM and cumulative fluid balance, and (3) the association between IAM trajectories and 30-day mortality. By integrating physiological reasoning with pragmatic monitoring tools, the study sought to determine whether IAM may serve as a clinically actionable marker of fluid responsiveness and an early prognostic indicator in sepsis management outside the ICU setting.

## 2. Methods

### 2.1. Study Design and Setting

This is a prospective observational study with repeated measures conducted at the IMCU of Alto Vicentino Hospital, Santorso, Italy, from 1 October 2022 to 1 February 2025. The IMCU at Alto Vicentino Hospital is a high-intensity care unit consisting of 12 beds, specifically designed for the stabilization of critically ill medical patients admitted from the Emergency Department (ED) or other general wards presenting with single or multiple acute organ failures who do not require immediate organ replacement therapy (e.g., invasive mechanical ventilation or continuous renal replacement therapy). The primary aim of this setting is to prevent clinical deterioration and avoid subsequent admission to the ICU. The IMCU is equipped with advanced multiparametric monitoring systems and provides high-intensity treatments, facilitating rapid patient turnover, with an average length of stay between 72 and 96 h aimed at stabilizing acute conditions. The unit can manage multi-organ dysfunction syndromes through non-invasive ventilation therapies, continuous invasive monitoring, and pharmacological hemodynamic support.

### 2.2. Patients

All patients admitted from the ED for community-acquired sepsis were considered. The diagnosis of sepsis was established according to the latest guidelines, defined as a suspected or confirmed infection as determined by the attending physician and a Sequential Organ Failure Assessment (SOFA) score ≥ 2 attributable to the infection.

Upon arrival at the IMCU, informed consent was requested from all potentially eligible patients. If the patient was unable to provide consent, it was obtained from family members. Patients who did not provide consent were excluded from the study.

Patients were also excluded if they met one or more of the following criteria: (1) Age below 18 years; (2) Pregnancy; (3) ED length of stay exceeding 6 h prior to IMCU transfer; (4) Administration of fluid volume exceeding 500 mL within the last 3 h in the ED; (5) Initiation of vasopressor therapy in the ED prior to IMCU admission (institutional criteria mandate ICU admission for patients with vasopressor-dependent shock); (6) Infection associated with a surgical procedure performed within 30 days prior to ED presentation; (7) Confirmed or suspected major bleeding, as assessed by the IMCU physician; (8) Low likelihood of survival within the first 24 h post-admission; (9) Terminal malignancy with an expected survival of less than 3 months, based on clinical judgment incorporating medical history, physical examination, functional status, and presenting symptoms; (10) Admission to IMCU from hospital wards other than the ED.

### 2.3. Variables

At enrollment, demographic data (age, sex), anthropometric measurements (height, weight, and body mass index), previous comorbidities (Charlson Comorbidity Index [CCI]), and baseline clinical characteristics (SOFA, NEWS, and APACHE II scores) were recorded for all patients. Laboratory measurements included plasma concentrations of albumin, haemoglobin, and haematocrit, obtained daily for up to five days following admission. These values were used to calculate IAM, expressed in grams, as the product of plasma albumin concentration and estimated plasma volume. Plasma volume was derived using validated anthropometric formulas and adjusted for systemic haematocrit using a correction coefficient (0.91).

Changes in IAM over time were used to calculate the NAL, defined as the difference in IAM between two consecutive timepoints. Net albumin loss (NAL) was interpreted as an indicator of albumin loss into the interstitial compartment. Urinary albumin losses were considered negligible.

Fluid administration data were extracted from electronic medical records and included the total volume of crystalloids administered (oral and intravenous). These were recorded as both interval (between timepoints) and cumulative volumes. All patients received urinary catheterization for hourly diuresis monitoring. Cumulative fluid balance (CFB) was calculated as the difference between total input and output volumes, normalized to baseline body weight and expressed as a percentage.

### 2.4. Study Protocol

Plasma concentrations of albumin, haemoglobin, and haematocrit were measured according to a standardized protocol, for a maximum period of five days from enrolment, using blood samples. The first blood sample was drawn at the time of IMCU admission, immediately after arterial line placement. Subsequent samples were collected daily at 08:00 a.m., up to and including the fifth day.

Values obtained for albumin, haemoglobin, and haematocrit at each timepoint were used to calculate the point-in-time IAM. Urinary albumin losses were considered negligible for analytical purposes. All data related to intravenous fluid administration were systematically recorded in the electronic medical record. Blood transfusions, if performed, were also documented; haemoglobin and haematocrit values were reassessed after each transfusion, and transfusion events were recorded accordingly.

Volume management of enrolled patients was conducted in accordance with the Surviving Sepsis Campaign recommendations, supplemented by the clinical judgment of the treating physician. All patients received exclusively crystalloid solutions (normal saline or Ringer’s lactate); the use of colloids, including human albumin, was excluded by protocol and was not employed throughout the study. The total volume of administered fluids (intravenous or oral) was recorded as input (mL) at each scheduled timepoint. These administrations were categorized into two distinct variables: (1) volumes infused between two consecutive assessments, and (2) cumulative volumes since enrolment.

Upon IMCU admission, all patients underwent urinary catheterization with hourly monitoring. Urinary output was recorded in milliliters, differentiating values between two consecutive timepoints and cumulative outputs. Based on these data, the cumulative fluid balance (CFB) was calculated and expressed as a percentage using the following formula:

$$CFB\left(\%\right)=\frac{Cumulative\;input\left(mL\right)-Cumulative\;output\left(mL\right)}{Initial\;body\;weight\left(kg\right)}\times 100$$

The CFB was used as a surrogate marker of the effects of fluid therapies on patient volume status and was subsequently compared with IAM.

All medical personnel involved underwent dedicated training sessions prior to the start of the study to ensure uniform adherence to the therapeutic protocol. Blood transfusions were administered according to predefined and documented clinical criteria.

### 2.5. Calculation of Intravascular Albumin Mass (IAM) and Definition of Net Albumin Leakage (NAL)

The IAM represents the total quantity of albumin present in the plasma compartment at a specific time. The NAL is defined as the net loss of albumin from the intravascular to the interstitial compartment between two consecutive evaluations.

NAL calculation is based on variations in IAM over time, accounting for concurrent changes in blood volume and protein concentrations.

IAM, expressed in grams (g), is calculated as the product of the plasma albumin concentration (in g/L) and the estimated plasma volume (in liters, L):

IAM=alb×PV

Plasma volume (PV) is derived from total blood volume (BV) and systemic Hct, adjusted for erythrocyte distribution between microcirculation and macrocirculation using the following correction factor:

$$PV=\left(1-Hct\times 0.91\right)\times BV$$

The factor 0.91 is a correction coefficient reflecting that systemic Hct overestimates the actual erythrocyte volume throughout the whole body. Total BV can be estimated using common validated anthropometric formulas based on gender, height, and body weight (e.g., Nadler’s formula). Alternatively, an indirect method to validate plasma volume estimation involves calculating the intravascular hemoglobin mass (MHb), derived as:

MHb = BV × Hb

where BV is the total blood volume and Hb is the hemoglobin concentration (g/L). Assuming a stable red blood cell mass in the absence of bleeding or transfusion, MHb allows for back-calculation of plasma volume via:

PV = (MHb/Hb) × (1 − Hct × 0.91)

This indirect derivation provides a physiological cross-check of plasma volume, leveraging the presumed constancy of MHb over time under stable hematologic conditions. However, in septic patients, fluctuations in hematocrit or hemoglobin due to hemodilution, hemoconcentration, or transfusions may introduce estimation biases. To mitigate this, we reassessed hematologic parameters following any transfusion events per protocol.

The NAL represents the net change in albumin mass within the vascular compartment between two consecutive timepoints, assuming that these changes result from albumin extravasation minus lymphatic return:

$$NAL={IAM}_{after}-{IAM}_{before}$$

A proportionally greater decrease in IAM compared to concurrent changes in MHb (assumed stable in the absence of bleeding or transfusion) indicates net loss toward the extravascular compartment. These equations were applied at each sampling timepoint, enabling dynamic assessment of IAM across evaluations and throughout the first five days of hospitalization. In this formulation, a positive NAL value reflects a net loss of albumin from the intravascular space between two measurements, while a negative NAL value would indicate a net gain.

### 2.6. Outcomes

The primary endpoint of the study was 30-day mortality from the time of enrolment. Mortality status was ascertained by consulting municipal death registries. The secondary endpoint was to evaluate the association between cumulative fluid balances and daily IAM serum levels.

### 2.7. Statistical Analysis

Continuous variables were described as mean and standard deviation (SD) or as median and interquartile range (IQR, 25th–75th percentile), as appropriate based on their distribution. Categorical variables were reported as absolute frequencies and percentages.

Univariate comparisons were performed using Student’s *t*-test or Mann–Whitney U test, and chi-square or Fisher’s exact tests for categorical data, depending on variable type and distribution. Correlations between IAM and continuous variables were assessed using Pearson’s or Spearman’s correlation coefficients, based on data normality.

To investigate the association between IAM trajectories and CFB, we employed repeated-measures Generalized Estimating Equation (GEE) models with an identity link and Gaussian distribution. This modeling approach accounts for within-subject correlation due to longitudinal observations. Models were adjusted for key clinical covariates, including age, BMI, comorbidities (Charlson Comorbidity Index), and clinical severity scores (SOFA and APACHE II). The effect of CFB was expressed as the cumulative daily percentage change, evaluated in 1% increments.

To assess potential effect modification by clinical severity, an interaction term between CFB and SOFA score was added to the GEE model, while maintaining the same set of adjustment covariates. The clinical relevance of the interaction was interpreted based on both statistical significance and the direction of the estimated coefficients.

To evaluate the relationship between IAM dynamics and 30-day mortality, we fitted a GEE model with a logit link and binomial distribution. Mortality was treated as a binary outcome, and time was modeled as a longitudinal factor. The model was adjusted for the same covariates described above, and CFB was included to assess its independent and potentially modifying effect on mortality risk.

All statistical analyses were performed using Stata version 16.1 (StataCorp LLC, College Station, TX, USA). Two-tailed *p*-values < 0.05 were considered statistically significant.

### 2.8. Ethical Consideration

The study was approved by the local ethics committee (Clinical Trial Ethics Committee ULSS 8, Berica-Vicenza, Italy; approval number: 19814) and was conducted in accordance with the ethical principles for medical research involving human subjects as defined by the Declaration of Helsinki. Informed consent was obtained from all eligible patients. In cases where the patient was unable to provide consent, it was sought from the next of kin or legal guardian.

## 3. Results

**Figure 1:**
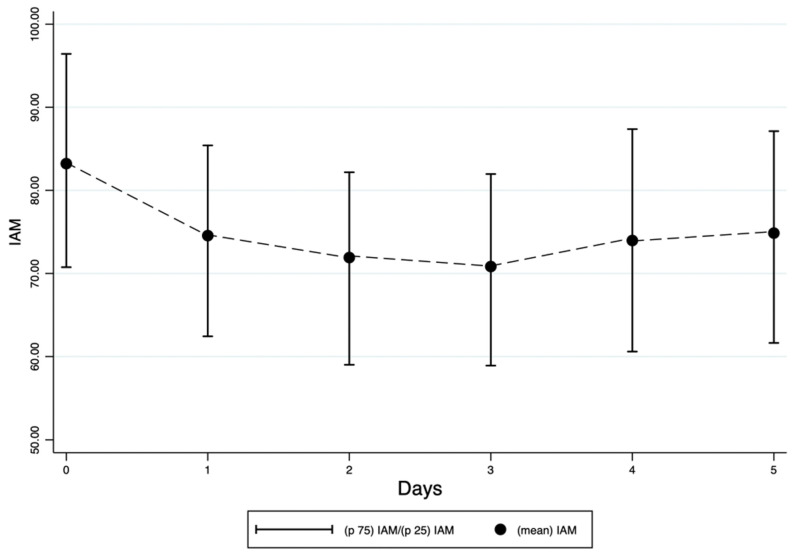
Temporal trends of mean IAM values in the enrolled patients.

A total of 247 patients were enrolled in the study. The mean IAM at the time of enrollment was 83.2 (SD 20.6). IAM decreased from a mean of 83.2 (SD 20.6) at enrollment to 74.8 (SD 20.2) at the fifth evaluation (Figure 1).

The mean NAL value at the last available evaluation for the entire cohort was 8.9 (SD 14.5). The mean CFB percentage change from baseline (CFB%) to the last evaluation was +1.42% (SD 5.46). The percentage CFB at the last evaluation showed a positive correlation with NAL at the last evaluation (Spearman’s rho = 0.257, *p* < 0.001). The correlations between IAM and CFB at each evaluation point are detailed in Table 1.

Pearson correlation analyses revealed a statistically significant association between cumulative fluid balance and IAM on all evaluated days, with negative coefficients ranging from −0.175 to −0.292 (*p* < 0.01). Simultaneously, cumulative NAL demonstrated inverse correlations with IAM and positive correlations with fluid balance, all of which were statistically significant across all timepoints (*p* < 0.001). The strongest correlations were observed during the first three days, with a slight attenuation thereafter (Table 1).

In the population-average GEE repeated-measures model, adjusted for comorbidities (CCI), clinical severity (SOFA, APACHE II), BMI, and age, cumulative fluid balance was significantly associated with changes in IAM across reassessments, with a coefficient of −1.08 (standard error 0.13; *p* < 0.001; 95% CI: −1.33 to −0.84). Specifically, each 1% increase in cumulative fluid balance from one day to the next was associated with an average decrease of 1.08 g in circulating albumin mass, independent of age, clinical condition, and comorbidity burden (Table 2).

In the subsequent GEE repeated-measures model including an interaction term for SOFA score, no significant modification of the effect of cumulative fluid balance on IAM was observed as a function of SOFA score (interaction coefficient = −0.0048; *p* = 0.940).

In the study cohort, 14.9% of patients (37/247) died. The characteristics of the deceased patients are listed in Table 3.

The mean IAM at enrolment was 85.1 (SD 20.8) in patients who survived at 30 days, compared with 72.6 (SD 15.4) in those who died within 30 days (*p* < 0.001). This difference was even more pronounced in non-survivors across all subsequent reassessment timepoints (Table 4).

**Figure 2:**
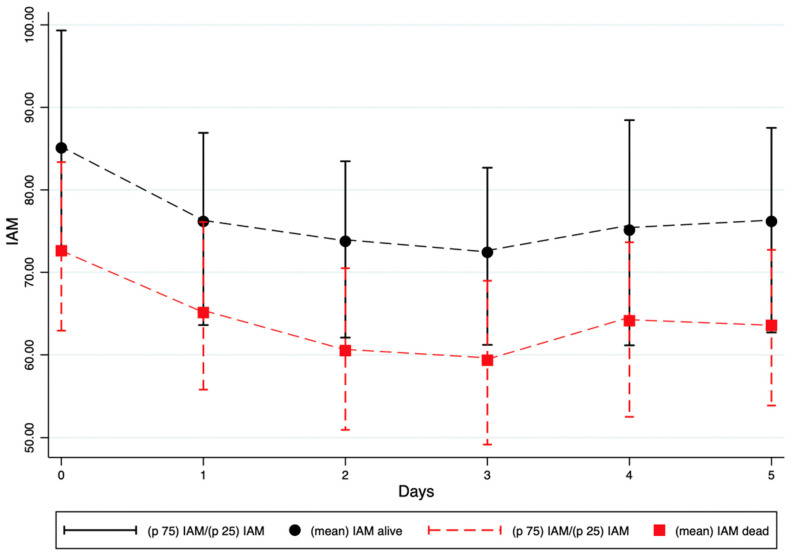
Temporal trends of IAM values in 30-day survivors and non-survivors.

At enrolment, non-survivors already exhibited significantly lower IAM values compared with survivors (72.6 vs. 85.1, *p* < 0.001). IAM continued to decline in non-survivors, reaching a nadir of 59.3 at the third reassessment. Throughout all subsequent timepoints, survivors consistently showed higher IAM values compared to non-survivors. These differences remained statistically significant up to the fifth measurement (*p*-values ranging from <0.001 to 0.007) (Figure 2).

The mean NAL value from admission to the last available assessment was 8.1 g (SD 14.1) in the overall cohort, compared with 13.7 g (SD 16.1) in patients who died within 30 days (*p* = 0.032) (Table 3). Patients who died were significantly older (76.5 ± 10.2 years vs. 69.6 ± 13.5 years, *p* = 0.037) and had higher average scores for both NEWS (6.8 ± 3.2 vs. 4.7 ± 3.1, *p* < 0.001) and SOFA (5.2 ± 2.8 vs. 3.9 ± 1.7, *p* < 0.001). No significant differences were found regarding sex, BMI, plasma volume, or APACHE score (Table 3).

In a repeated-measures GEE model with logit link, IAM remained an independent predictor of 30-day mortality (Table 5). Specifically, each 1-g increase in IAM was associated with a 2.1% reduction in the risk of death (OR = 0.98; 95% CI: 0.97–0.99; *p* < 0.001), after adjustment for age, comorbidities (CCI), clinical severity (SOFA, NEWS), and cumulative fluid balance (Table 5). In a subsequent repeated-measures GEE model including an interaction term with SOFA score, IAM remained an independent predictor of mortality (coef. = −0.0431; *p* = 0.002). The interaction term between IAM and SOFA showed a coefficient of 0.0045 (*p* = 0.066), suggesting a potential modulation of IAM’s effect by clinical severity, though this did not reach statistical significance. To explore whether serum albumin alone could similarly predict mortality, we conducted an additional repeated-measures GEE model replacing IAM with serum albumin level, using the same covariates. As shown in Supplementary Table S1, serum albumin was also significantly associated with 30-day mortality (*p* < 0.001).

## 4. Discussion

This prospective study of patients with community-acquired sepsis admitted to a high-intensity Intermediate Care Unit (IMCU) identified a progressive decline in Intravascular Albumin Mass (IAM) during the initial five days of hospitalization. Non-survivors demonstrated significantly lower IAM values at baseline and throughout all reassessments, with the most pronounced decline occurring within the first 72 h. A consistent inverse relationship was observed between IAM and cumulative fluid balance, while NAL was positively correlated with fluid accumulation, suggesting a physiological link between albumin redistribution and capillary leak. After adjusting for age, comorbidities, and severity scores, lower IAM remained independently associated with higher 30-day mortality. None of the patients in this study were in vasopressor-requiring shock at enrollment, as such patients were triaged directly to the ICU. The association between lower IAM at enrollment and mortality reflects underlying illness severity rather than shock status. This is supported by higher SOFA and NEWS scores in non-survivors, indicating more severe organ dysfunction at baseline. These findings support the potential of IAM as a dynamic, physiology-based marker of fluid responsiveness and early prognosis in sepsis, particularly in settings outside the intensive care unit.

The observed association between declining IAM and adverse outcomes underscores the potential of IAM as a clinically useful, real-time marker of vascular permeability and fluid responsiveness in sepsis. Unlike static serum albumin concentrations, IAM reflects the actual intravascular albumin pool by accounting for concurrent changes in plasma volume and hemoconcentration [16,17]. This mass-based approach provides a more accurate estimate of effective oncotic pressure and vascular protein loss, both of which are central to the pathophysiology of capillary leak syndrome [18]. As IAM can be derived from routinely available laboratory parameters, such as albumin, hemoglobin, and hematocrit, it offers a practical, non-invasive tool to monitor endothelial function and the evolving intravascular status of septic patients. In settings such as intermediate care, where invasive monitoring is often limited, serial IAM assessments may help identify patients at risk of fluid overload or impending deterioration and support individualized resuscitation strategies. While serum albumin has long been recognized as a reliable prognostic biomarker in sepsis, its interpretation is often limited by confounding effects of hemodilution, fluid overload, and redistribution. In our additional analysis (Supplementary Table S1), serum albumin concentration was independently associated with 30-day mortality, confirming its known prognostic value. However, the aim of this study was not to replace albumin with IAM for mortality prediction, but rather to characterize IAM as a dynamic, physiology-based index that reflects the actual intravascular albumin pool over time [16,17].

Unlike serum albumin, which represents a static concentration at a single time point, IAM incorporates dynamic changes in plasma volume and provides a more direct estimate of the oncotic capacity of the vascular compartment. This distinction is particularly relevant in sepsis, where intravascular volume status and capillary integrity are in constant flux. By capturing the interaction between albumin concentration and estimated plasma volume, IAM allows for a more nuanced understanding of albumin kinetics in response to fluid resuscitation, capillary leak, and redistribution phenomena.

Therefore, IAM should not be viewed solely as a prognostic biomarker, but rather as a complementary tool for assessing the evolution of fluid dynamics, endothelial function, and vascular protein loss in septic patients. Future studies may investigate whether integrating IAM with serum albumin measurements enhances the clinical management of sepsis-related fluid imbalance and guides individualized resuscitation strategies.

While established clinical severity scores such as SOFA, NEWS, and APACHE II remain essential for early risk stratification in sepsis [19], they do not directly capture the dynamic processes of vascular permeability and protein redistribution. In this context, IAM provides distinct and physiologically grounded information that complements these scores rather than replaces them. The findings of this study suggest that IAM may detect early vascular compromise and ongoing capillary leak before these changes manifest as overt organ dysfunction or score deterioration. Notably, IAM retained independent prognostic significance for 30-day mortality even after adjustment for SOFA and other clinical indices, supporting its additive value. Integrating IAM with conventional severity scoring could enhance early identification of high-risk patients and refine decision-making regarding fluid resuscitation, monitoring intensity, and potential escalation of care.

The observed progressive decline in IAM within this cohort is consistent with established mechanisms of impaired vascular protein homeostasis in sepsis. Under physiological conditions, transient increases in lymphatic return, known as “interstitial washdown,” can partially mitigate albumin loss by returning protein-rich lymph to the vascular space. Hahn and Dull [12] quantified this mechanism during crystalloid infusion, demonstrating that albumin recruitment from the interstitium can elevate plasma albumin levels by 0.3–1.0 g/L, primarily through accelerated lymphatic flow. However, this compensatory process is rapidly exhausted, and its efficacy relies on intact lymphatic function and stable interstitial matrix integrity. In sepsis, inflammatory mediators such as TNF-α and nitric oxide disrupt lymphatic pumping and reduce interstitial hydrostatic pressure, effectively halting washdown [20]. This pathophysiological failure likely contributes to the persistent albumin loss and rising NAL observed in non-survivors in the present study. Thus, the declining IAM trajectory may not merely reflect capillary leak but also the collapse of compensatory protein refill mechanisms, underscoring its value as an early indicator of endothelial and lymphatic dysfunction in septic patients.

The present findings suggest that decreases in IAM are more sensitive to early fluid redistribution and vascular permeability changes than cumulative fluid balance (CFB) alone. While CFB reflects the net volumetric input–output over time, it does not differentiate between effective intravascular volume expansion and third-space fluid accumulation [21]. In contrast, IAM incorporates both plasma albumin concentration and dynamic estimates of plasma volume, capturing subtle shifts in albumin compartmentalization driven by capillary leak. Notably, in this cohort, IAM decline was evident even in patients with modest or near-neutral fluid balances, indicating that albumin extravasation may precede or occur independently of overt fluid overload. Furthermore, IAM correlated inversely with CFB across all timepoints, while NAL was positively correlated, suggesting that ongoing vascular albumin loss may act as a more immediate and pathophysiologically grounded marker of fluid misdistribution. These observations imply that IAM decline may serve as an early signal of ineffective fluid retention and impending edema formation, offering higher sensitivity than gross fluid balance in detecting microvascular dysfunction during sepsis resuscitation.

The calculation of IAM reflects the interaction between plasma albumin concentration and PV, which vary during sepsis management. IAM could increase through rising albumin concentrations or PV contraction. Conversely, PV expansion from fluid administration could dilute albumin concentrations and reduce IAM decline, even during transcapillary albumin loss. In our cohort, PV expansion with declining albumin concentrations resulted in a net reduction in IAM.

We calculated PV at each time point using hematocrit-adjusted blood volume estimates to incorporate PV changes into IAM and NAL assessments. NAL, defined as the change in IAM between consecutive days, reflects both albumin extravasation and intravascular volume shifts. In patients with significant PV expansion, NAL may reflect dilutional effects rather than capillary leak. Therefore, IAM and NAL should be interpreted as markers of albumin redistribution influenced by vascular permeability and volume status. Future studies using direct PV measurements or tracer-based albumin kinetics could disentangle these components.

We recognize that the term “Net Albumin Loss” may imply directional ambiguity. As defined in this study, positive NAL represents a decrease in IAM, corresponding to net loss of albumin from the vascular space, while negative values would indicate gains, which were infrequent. Given the sepsis context and our pathophysiologic focus on capillary leak, we retained this terminology while clarifying its operational meaning.

The relationship between CFB and IAM appeared consistent across the clinical severity spectrum. The absence of a significant interaction between CFB and SOFA score suggests that the association between fluid accumulation and IAM decline is independent of initial illness severity. This finding implies that even in less critically ill patients, early positive fluid balance may contribute to albumin redistribution and potential capillary leak, reinforcing the importance of fluid stewardship across the full spectrum of sepsis severity.

The current findings are corroborated by emerging evidence regarding the pathophysiology of albumin kinetics, particularly in the context of capillary leak and altered fluid distribution in sepsis. Hahn and colleagues [22] demonstrated that hyperoncotic albumin solutions, such as 20% albumin, are more effective than crystalloids in maintaining plasma volume expansion in hypotensive states, due to their capacity to recruit fluid from the interstitium even at low mean arterial pressures. This observation aligns with the inverse association between IAM and cumulative fluid balance identified in the present study, suggesting that a decline in albumin mass may indicate ongoing vascular leak rather than intravascular volume depletion. Furthermore, volume kinetic modeling of 20% albumin in volunteers and postoperative patients revealed that neither inflammation nor elevated glycocalyx degradation markers significantly influenced albumin elimination kinetics, and that capillary leakage remained stable despite systemic inflammatory stimuli [23]. These findings challenge prevailing assumptions of universally increased albumin leakage in septic states and support IAM as a more specific marker of fluid responsiveness and vascular compartment shifts. Notably, structural assessments of the endothelial glycocalyx have shown inconsistent correlation with plasma biomarkers, underscoring the complexity of using syndecan-1 or heparan sulfate alone as indicators of vascular permeability [24]. Collectively, these insights underscore the potential of IAM as a dynamic, physiologically grounded index of capillary barrier function and fluid distribution in early sepsis management.

The present analysis further suggests that the protective association between higher intravascular albumin mass and survival may be modulated by clinical severity. Although the interaction term between IAM and the SOFA score did not reach statistical significance, the observed trend toward attenuation of the albumin effect at higher SOFA levels is pathophysiologically plausible. In states of pronounced endothelial injury, reflected by elevated SOFA scores, capillary barrier dysfunction may be so advanced that the capacity of albumin to exert oncotic pressure and maintain intravascular volume becomes compromised. This interpretation is consistent with experimental and kinetic data showing that severe inflammatory states reduce the efficacy of albumin in sustaining plasma volume expansion due to overwhelming transcapillary leakage [25]. Thus, while intravascular albumin mass appears to function as a robust prognostic marker across the cohort, its clinical utility as a therapeutic guide may depend on the underlying degree of endothelial integrity.

The calculation of plasma volume in this study, derived from anthropometric estimates of total blood volume and adjusted for hematocrit using a correction coefficient, presents both practical strengths and methodological limitations. On the one hand, this approach enables non-invasive, repeatable estimation of PV using readily available clinical data, namely, height, weight, sex, and laboratory measurements of hematocrit [26]. The use of the 0.91 correction factor accounts for the known discrepancy between systemic hematocrit and true intravascular red cell distribution, enhancing physiologic accuracy. This method also facilitates dynamic tracking of IAM without the need for exogenous tracers, allowing for repeated assessment across timepoints in real-world settings such as intermediate care. However, this approach is not without drawbacks. First, the formula assumes stable red blood cell mass and homogeneous distribution of hematocrit, which may not hold in septic patients experiencing hemodilution, hemoconcentration, or transfusions [27]. Second, body size-based blood volume estimation may misrepresent true PV in patients with obesity, cachexia, or capillary leak syndromes [28]. Lastly, small errors in hematocrit measurement or anthropometric input can propagate and magnify uncertainties in IAM calculations. While sufficient for cohort-level trends, these limitations should be acknowledged when interpreting individual-level PV and IAM estimates in clinical decision-making.

The clinical utility of IAM and NAL is best suited for monitoring and fluid stewardship phases of sepsis care rather than early resuscitation. Since initial fluid resuscitation occurs within the first hours of presentation, real-time IAM and NAL data would not guide these early interventions. Instead, serial IAM measurements could provide insights during stabilization, helping clinicians assess vascular integrity, identify fluid overload risks, and tailor fluid therapy or initiate de-resuscitation. Point-of-care methods or automated calculations from routine labs could enhance the timeliness of assessments to inform clinical decisions.

Future research should focus on validating the prognostic and physiological utility of IAM in larger, multicenter cohorts across diverse healthcare settings, including emergency departments, intermediate care units, and intensive care units. External validation would elucidate the generalizability of IAM trends and thresholds concerning fluid resuscitation outcomes and mortality. Furthermore, integrating IAM dynamics into structured fluid stewardship protocols, potentially in conjunction with capillary leak indices or bioimpedance-based assessments, may facilitate real-time tailoring of fluid therapy. Given that IAM can be derived from standard laboratory data, there is potential for developing automated bedside calculators or electronic decision-support tools that monitor IAM and NAL longitudinally. Such tools could aid clinicians in identifying early signs of ineffective resuscitation or evolving vascular dysfunction, thereby supporting timely intervention before irreversible organ damage occurs.

Several limitations should be considered when interpreting these findings. First, the single-center design may limit the external validity of the results, particularly concerning regional practice patterns, patient demographics, and fluid management protocols. Second, the strict exclusion criteria, such as prolonged emergency department stays, early vasopressor use, or recent surgery, were necessary to isolate community-acquired sepsis in a uniform clinical context but may reduce generalizability to broader septic populations. Third, the study was observational and unblinded, introducing the potential for observer or treatment bias, especially in clinical decisions regarding fluid administration. Although multivariable models were employed to adjust for key confounders, residual confounding cannot be entirely excluded. Factors such as undetected bleeding, unmeasured nutritional status, hepatic synthetic function, or subclinical albumin supplementation may have influenced IAM independently of fluid dynamics. We acknowledge that the estimation of PV and MHb is inherently indirect and subject to error, particularly in sepsis, where hemoconcentration, hemodilution, and altered erythrocyte dynamics can occur. While our protocol included reassessment after transfusions, direct measurement of red cell mass or plasma volume (e.g., using radioisotope dilution) would provide higher precision but was not feasible in this clinical setting. A decline in Hb and Hct due to inflammation or blood loss could influence PV calculation and IAM. While our protocol accounted for hematologic parameters at each time point, ongoing anemia may introduce variability in PV estimates that could affect IAM trajectories. This limitation should be addressed in future studies analyzing anemia progression and IAM kinetics.

The observed decrease in IAM likely reflects not only albumin redistribution due to capillary leak but may also be influenced by reduced hepatic albumin synthesis and increased degradation in the context of sepsis-induced catabolism. Although our study did not assess these mechanisms directly, their contribution cannot be excluded and warrants investigation in future studies using specific biomarkers or tracer methodologies. Our findings align with earlier evidence linking hypoproteinemia to adverse sepsis outcomes. Mangialardi et al. [29] showed that low total serum protein levels strongly predict acute respiratory distress syndrome, fluid retention, and mortality in sepsis patients, independent of disease severity. While their study focused on total serum protein’s role in oncotic pressure and fluid shifts, our focus on IAM offers specific insights into the principal oncotic protein’s dynamics. This specificity may refine understanding of vascular barrier dysfunction and protein redistribution contributing to poor sepsis outcomes.

## 5. Conclusions

In patients with community-acquired sepsis admitted to an intermediate care unit, IAM exhibited a progressive decline during the initial phase of hospitalization and was independently correlated with cumulative fluid balance and 30-day mortality. The temporal progression of IAM, rather than absolute serum albumin concentrations or net fluid balance alone, may indicate underlying capillary leak and the failure of compensatory albumin refill mechanisms. These findings suggest that IAM could serve as a physiologically informative and clinically accessible marker of fluid distribution and vascular dysfunction in sepsis. Future research should assess the prognostic value of IAM in larger, multicenter cohorts and explore its integration into fluid stewardship strategies to guide individualized resuscitation and improve outcomes in acute care settings.

## Figures and Tables

**Table 1:** Spearman correlations between the IAM and NAL with CFB from baseline to Day 5.

Correlations	Cumulative Fluid Balance	*p*-Value
IAM Day 1	−0.292	<0.001
NAL from baseline to Day 1	0.254	0.001
IAM Day 2	−0.276	<0.001
NAL from baseline to Day 2	0.257	<0.001
IAM Day 3	−0.259	<0.001
NAL from baseline to Day 3	0.311	<0.001
IAM Day 4	−0.175	0.008
NAL from baseline to Day 4	0.186	0.005
IAM Day 5	−0.269	<0.001
NAL from baseline to Day 5	0.251	<0.001

**Table 2:** Population-averaged GEE model of IAM, adjusted for clinical covariates.

IAM GEE Model			
Variables	Coefficent	95% CI	*p*-Value
CFB	−1.083	−1.331–−0.835	<0.001
CCI	−0.330	−0.814–0.154	0.181
SOFA	−0.514	−1.057–0.027	0.063
APACHE II	−0.352	−0.604–−0.100	0.006
BMI	−886.865	−1011.862–−761.867	<0.001
Age	−0.147	−0.243–−0.050	0.003

**Table 3:** Baseline demographic, anthropometric, and clinical characteristics of the study cohort stratified by 30-day survival status.

Variables	Total	Alive at 30 Days	Dead at 30 Days	*p*-Value
Patients, n (%)	247 (100)	210 (85.1)	37 (14.9)	
Age, years, mean (SD)	70.6 (13.3)	69.6 (13.5)	76.5 (10.2)	0.037
Sex, n (%)				0.466
Male	150 (60.7)	125 (59.5)	25 (67.6)	
Female	97 (39.3)	85 (40.5)	12 (32.4)	
Anthropometric data				
Height, m^2^, mean (SD)	1.71 (0.11)	1.72 (0.09)	1.69 (0.11)	0.213
Weight, kg, mean (SD)	74.5 (15.6)	75.1 (16.1)	70.9 (12.6)	0.135
BMI, mean (SD)	25.3 (4.7)	25.4 (4.9)	24.7 (3.7)	0.356
Plasma Volume, mean (SD)	3.2 (0.5)	3.2 (0.5)	3.1 (0.5)	0.529
NEWS score, point, mean (SD)	5.1 (3.2)	4.7 (3.1)	6.8 (3.2)	<0.001
SOFA score, point, mean (SD)	4.1 (1.9)	3.9 (1.7)	5.2 (2.8)	<0.001
APACHE II score, point, mean (SD)	21.6 (4.1)	21.5 (4.2)	22.3 (3.8)	0.2697
IAM, mean (SD)	83.2 (20.6)	85.1 (20.8)	72.6 (15.4)	<0.001
NAL from baseline to the last available assessment, mean (SD)	8.9 (14.5)	8.1 (14.1)	13.7 (16.1)	0.032

**Table 4:** Temporal evolution of IAM across study timepoints, stratified by 30-day survival status.

Timepoint	n (Patients)	IAM, Mean (SD)	Alive at 30 Days, Mean (SD)	Dead at 30 Days, Mean (SD)	*p*-Value
**Baseline**	247	83.2 (20.6)	85.1 (20.8)	72.6 (15.4)	<0.001
**Day 1**	246	74.6 (18.6)	76.2 (18.9)	65.1 (13.7)	<0.001
**Day 2**	244	71.9 (18.6)	73.7 (18.4)	60.5 (15.8)	<0.001
**Day 3**	238	70.8 (18.8)	72.4 (18.6)	59.3 (15.1)	<0.001
**Day 4**	229	73.9 (19.1)	75.1 (19.1)	64.1 (15.7)	0.007
**Day 5**	220	74.8 (20.2)	76.2 (20.1)	63.6 (17.1)	0.004

**Table 5:** Population-averaged GEE model for 30-day mortality.

30-Day Mortality GEE Model			
Variables	Coefficent	95% CI	*p*-Value
IAM	−0.020	−0.030–−0.009	<0.001
CFB	0.137	0.087–0.188	<0.001
CCI	0.133	0.052–0.214	0.001
SOFA	0.114	0.016–0.213	0.023
NEWS	0.145	0.079–0.212	<0.001
Age	0.024	0.003–0.046	0.022

## Data Availability

Data available on request due to privacy/ethical restrictions.

## Supplementary Materials

The following supporting information can be downloaded at: https://www.mdpi.com/article/10.3390/jcm14155255/s1, Table S1: GEE model for 30-day mortality using serum albumin level (g/dL) as the main predictor, adjusted for age, cumulative fluid balance, CCI, SOFA, and NEWS scores.
