# Supplementary material for: Temporal Decline in Intravascular Albumin Mass and Its Association with Fluid Balance and Mortality in Sepsis: A Prospective Observational Study
Source: J Clin Med. 2025 Jul 24;14(15):5255. doi: 10.3390/jcm14155255 (PMC12347051; doi:10.3390/jcm14155255)
Supplement: Supplementary file 1 [file jcm-14-05255-s001.zip › jcm-3742570-supplementary.pdf]

Supplementary Table 1: GEE model for 30-day mortality using serum albumin level (g/dL) as the main predictor, adjusted for age, cumulative fluid balance, CCI, SOFA, and NEWS scores.

**Variables**	**Coefficient**	**95% CI**	**p-value**
Albumin	-1.260	-1.762 - -0.757	<0.001
CFB	0.119	0.679 – 0.170	<0.001
CCI	0.114	0.032 – 0.196	0.006
SOFA	0.068	-0.032 – 0.169	0.182
NEWS	0.173	0.105 – 0.241	<0.001
Age	0.030	0.009 – 0.052	0.005
